# Gene expression profiling of subcutaneous adipose tissue in morbid obesity using a focused microarray: Distinct expression of cell-cycle- and differentiation-related genes

**DOI:** 10.1186/1755-8794-3-61

**Published:** 2010-12-23

**Authors:** Sara Rodríguez-Acebes, Nuria Palacios, José I Botella-Carretero, Nuria Olea, Lorena Crespo, Roberto Peromingo, Diego Gómez-Coronado, Miguel A Lasunción, Clotilde Vázquez, Javier Martínez-Botas

**Affiliations:** 1 Servicio de Bioquímica-Investigación, Hospital Ramón y Cajal, IRYCIS, Madrid, Spain; 2 CIBER de Fisiopatología de la Obesidad y Nutrición (CIBEROBN), Instituto de Salud Carlos III, Madrid, Spain; 3 Unidad de Cirugía de la Obesidad, Unidad de Nutrición Clínica y Dietética, Servicio de Endocrinología y Nutrición, Hospital Ramón y Cajal, Madrid, Spain; 4 Unidad de Cirugía de la Obesidad, Servicio de Cirugía General Digestivo, Hospital Ramón y Cajal, Madrid, Spain; 5 Departamento de Bioquímica y Biología Molecular, Universidad de Alcalá, Alcalá de Henares, Spain

## Abstract

**Background:**

Obesity results from an imbalance between food intake and energy expenditure, which leads to an excess of adipose tissue. The excess of adipose tissue and adipocyte dysfunction associated with obesity are linked to the abnormal regulation of adipogenesis. The objective of this study was to analyze the expression profile of cell-cycle- and lipid-metabolism-related genes of adipose tissue in morbid obesity.

**Methods:**

We used a custom-made focused cDNA microarray to determine the adipose tissue mRNA expression profile. Gene expression of subcutaneous abdominal fat samples from 15 morbidly obese women was compared with subcutaneous fat samples from 10 nonobese control patients. The findings were validated in an independent population of 31 obese women and 9 obese men and in an animal model of obesity (Lep^ob/ob ^mice) by real-time RT-PCR.

**Results:**

Microarray analysis revealed that transcription factors that regulate the first stages of adipocyte differentiation, such as CCAAT/enhancer binding protein beta (C/EBPβ) and JUN, were upregulated in the adipose tissues of morbidly obese patients. The expression of peroxisome proliferator-activated receptor gamma (PPARγ), a transcription factor which controls lipid metabolism and the final steps of preadipocyte conversion into mature adipocytes, was downregulated. The expression of three cyclin-dependent kinase inhibitors that regulate clonal expansion and postmitotic growth arrest during adipocyte differentiation was also altered in obese subjects: p18 and p27 were downregulated, and p21 was upregulated. Angiopoietin-like 4 (ANGPTL4), which regulates angiogenesis, lipid and glucose metabolism and it is know to increase dramatically in the early stages of adipocyte differentiation, was upregulated. The expression of C/EBPβ, p18, p21, JUN, and ANGPTL4 presented similar alterations in subcutaneous adipose tissue of Lep^ob/ob ^mice.

**Conclusions:**

Our microarray gene profiling study revealed that the expression of genes involved in adipogenesis is profoundly altered in the subcutaneous adipose tissue of morbidly obese subjects. This expression pattern is consistent with an immature adipocyte phenotype that could reflect the expansion of the adipose tissue during obesity.

## Background

Obesity is the most common nutritional disorder in Western societies and is reaching epidemic proportions [1]. Obesity results from an imbalance between food intake and energy expenditure, which leads to an excess of white adipose tissue. Adipocytes are highly active endocrine cells that secrete many factors, including hormones, cytokines, growth factors, acute phase reactants, complement-related proteins, and extracellular matrix proteins, which can have an important impact on other organs and play a central role in the regulation of energy balance and insulin sensitivity [2]. Consequently, an excess of adipose tissue and adipocyte dysfunction are associated with an increased risk of developing type 2 diabetes mellitus, hypertension, dyslipidemia, stroke, cardiovascular disease, and a variety of cancers [3-5]. The metabolic risks associated with obesity correlate strongly with central adiposity, and subcutaneous truncal fat plays a major role in the pathophysiology of obesity complications, especially insulin resistance [6-8].

Excess adipose tissue is linked to the abnormal regulation of adipogenesis and adipocyte hypertrophy, and also to cell hyperplasia in more severe forms of obesity [9]. Adipocyte hyperplasia requires the recruitment and proliferation of preadipocytes present in the vascular stroma of adipose tissue [10]. Adipocyte differentiation is a complex process regulated by a number of transcriptional factors acting coordinately [11]. Most studies investigating adipocyte differentiation have been performed in murine preadipocyte cell lines and in animal models. In these models, adipocyte differentiation begins with a proliferative event known as clonal expansion, in which the cells undergo one or two rounds of cell division. They then exit the cell cycle and initiate terminal differentiation. Two families of transcription factors are the key regulators of this process and are responsible for activating the adipogenic gene program: the CCAAT/enhancer-binding proteins (C/EBPs) and peroxisome proliferator-activated receptors (PPARs) [12]. Clonal expansion and subsequent growth arrest are associated with changes in the expression of cyclin-dependent kinase inhibitors (CDKIs), which inhibit the cyclin-CDK complexes and thus control cell-cycle progression [13,14].

Much less is known about adipocyte differentiation in humans and its relation to development of obesity. The adipogenic program in human seems to be similar to that of murine cell lines [15], although in vitro human preadipocytes do not require clonal expansion to differentiate [16]. Genome-wide microarray analysis has been previously used in adipose tissue of human obese subjects to identify new candidate genes with abnormal expression, to explore the differences between distinct fat depots or to address the response to pharmaceutical or nutritional intervention [17-20]. In the present study, we sought to investigate the relation between obesity and adipocyte differentiation in vivo. For this purpose we analyzed the gene expression profile of abdominal subcutaneous adipose tissue in human morbid obesity using a custom-made focused cDNA microarray composed of 319 cDNA probes corresponding to genes involved in cell cycle, adipocyte differentiation and lipid metabolism [21]. We found that the expression of genes involved in adipogenesis, such as C/EBPβ, JUN, PPARγ, CDKN1A (p21), CDKN2C (p18) and ANGPTL4, is profoundly altered in the subcutaneous adipose tissue of morbidly obese subjects. This expression pattern could reflect the expansion of the adipose tissue during obesity.

## Results

### Patient characteristics

Subcutaneous fat samples from 15 morbidly obese women undergoing bariatric surgery were compared with subcutaneous fat samples from 10 nonobese control women. The patient characteristics are shown in Table 1. All the obese patients had BMI > 35 kg/m^2^, whereas the control subjects had BMI < 25 kg/m^2^. Fasting glucose levels were higher in morbidly obese patients, and two had a clinical diagnosis of diabetes (fasting glucose > 125 mg/dL), whereas all the control subjects had normal fasting glucose. Plasma triglyceride levels were significantly higher in obese patients and no differences were observed for cholesterol levels. As expected, systolic, diastolic, and mean blood pressures were higher in the obese individuals than in the controls (Table 1).

**Table 1:** Clinical and biochemical characteristics of women of the microarray study.

	Patients (n = 15)	Controls (n = 10)	P*
Current smokers, n (%)	3 (20)	0 (0)	0.237
Age (years)	49.6 ± 8.7	47.0 ± 17.4	0.770
Body mass index (Kg/m^2^)	48.1 ± 6.1	22.7 ± 3.4	< 0.001
Systolic blood pressure (mmHg)	137.7 ± 15.1	117.8 ± 9.7	0.001
Diastolic blood pressure (mmHg)	82.3 ± 10.7	64.4 ± 11.0	0.002
Mean blood pressure (mmHg)	100.8 ± 10.5	82.2 ± 10.3	< 0.001
Serum creatinine (mg/dL)	0.81 ± 0.08	0.78 ± 0.10	0.215
Aspartate aminotransferase (U/L)	21.1 ± 8.8	17.7 ± 2.5	0.174
Alanine aminotransferase (U/L)	27.9 ± 17.2	15.0 ± 5.4	0.007
Fasting glucose (mg/dL)	106.0 ± 22.6	93.0 ± 4.0	0.030
Total cholesterol (mg/dL)	204.9 ± 38.1	206.9 ± 36.8	0.728
Triglycerides (mg/dL)	150.0 ± 97.0	62.7 ± 21.9	0.001

Data are means ± SD unless otherwise stated.

*P value after Mann-Whitney U test for continuous data and Chi-square test or Fisher's exact test for categorical data as appropriate.

### Differential gene expression

**Figure 1:**
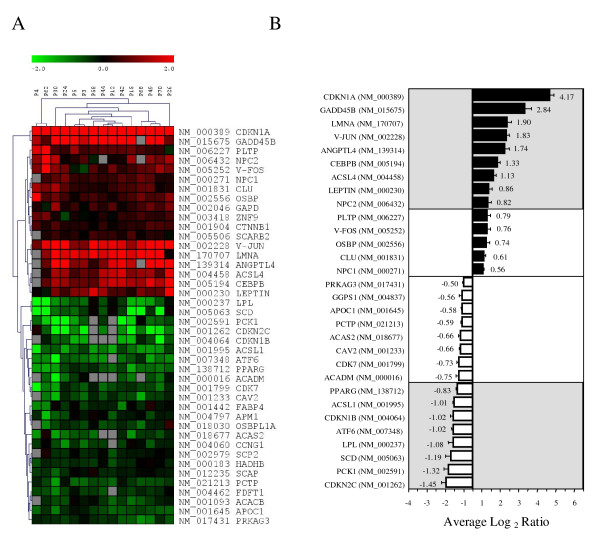
**Microarray gene expression profile of subcutaneous adipose tissues**. A) Hierarchical clustering analysis of the gene expression profiles of the subcutaneous adipose tissues of 15 morbidly obese patients. The results are represented as log_2 _ratios. Increasing red intensity denotes increased gene expression and increasing green intensity denotes decreased gene expression according to the scale bar at the top of the figure. The genes are grouped according to the similarity of their expression patterns using Euclidean distances and the average linkage clustering method. B) Upregulated and downregulated gene expression in the adipose tissues of morbidly obese subjects. The bars represent the mean gene expression ± SE determined with the microarray (n = 15). Grey areas represent the genes with average gene expression over or below the cut-off log_2 _ratio value of 0.8 or -0.8, respectively.

Subcutaneous fat gene expression profile in each of the obese patients was compared to a pool of nonobese control samples to minimize the influence of individual variability in controls. Of the 319 probes on the microarray, 42 remain after filtering for detectable expression, consistency of replicates and statistical significance (p < 0.001) (Figure 1A).

Of these, 9 were upregulated and 8 were downregulated in obese patients compared to the controls using a differential cut-off average log_2 _ratio of 0.8 (Figure 1B). Among these 17 genes, 4 were genes involved in cell-cycle regulation. The expression of cyclin-dependent kinase inhibitor 1A (CDKN1A/p21) and growth arrest and DNA-damage-inducible β (GADD45B) were upregulated, whereas that of cyclin-dependent kinase inhibitor 2C (CDKN2C/p18) and cyclin-dependent kinase inhibitor 1B (CDKN1B/p27) were downregulated in obese patients compared with control subjects (Figure 1). As regards genes involved in lipid metabolism, those encoding fatty-acid-CoA ligase long-chain 4 (ACSL4), leptin (LEP), and Niemann-Pick disease type C2 (NPC2) were upregulated, whereas those encoding stearoyl-CoA desaturase (SCD), lipoprotein lipase (LPL), and fatty-acid-CoA ligase long-chain 1 (ACSL1) were downregulated. The expression of transcription factors such as JUN and CCAAT/enhancer binding protein β (CEBPβ) was increased, whereas that of PPARγ was decreased. FOS expression tended to be upregulated, although did not meet the log_2 _cut-off ratio of 0.8 (Figure 1B). Phosphoenolpyruvate carboxykinase 1 (PCK1), a key enzyme of gluconeogenesis, was downregulated. Moreover, he expression of the cytoskeleton gene lamin A/C (LMNA) and the angiogenesis-related gene angiopoietin-like 4 (ANGPTL4) was also upregulated. Finally, the expression of the activating transcription factor 6 (ATF6), which is involved in the unfolded protein response, was lower than in control subjects.

**Figure 2:**
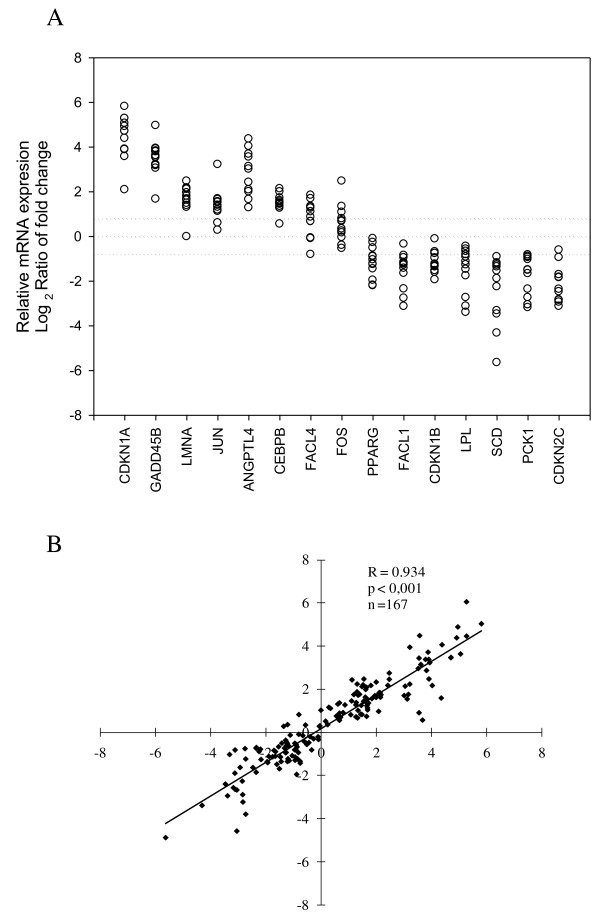
**Validation of the mRNA expression levels determined with the microarray by quantitative real-time RT-PCR and correlation of the data**. A) Individual values for mRNA expression by quantitative real-time RT-PCR. The real-time RT-PCR data are expressed as the log_2 _ratio of the fold change in the expression of each target gene using the relative quantification method by comparison with the expression of the housekeeping gene RPLP0 (Pfaffl, 2001). Each circle represents the mean of two replicates of the fold change between of each the obese sample and the pool of nonobese control samples (n = 12). B). Linear regression analysis of the results determined by microarray and real-time RT-PCR analyses. The x-axis represents the log_2 _ratios of the relative mRNA expression determined by real-time RT-PCR and the y-axis represents the log_2 _ratio obtained by microarray for each sample and gene analyzed (n = 167).

To validate the microarray results quantitatively, we selected 15 genes on the basis of their ratio ranking or their biological interest and we analyzed their expression by real-time RT-PCR in the same samples previously analyzed by the microarray. In all cases, a strong correspondence between the real-time RT-PCR results and the microarray data was observed (Figures 1B and 2A). Linear regression analysis of the log_2 _ratios obtained by microarray analysis and the log_2 _of the relative mRNA expression determined by real-time RT-PCR showed a highly statistically significant correlation (r = 0.934, *P *< 0.001, n = 167) (Figure 2B).

### Confirmation of the results in an independent set of morbidly obese patients

**Figure 3:**
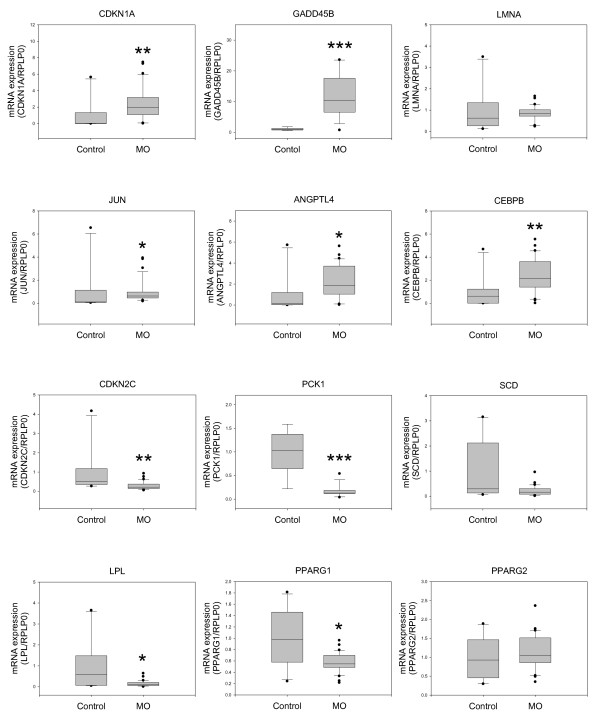
**Gene expression in the subcutaneous adipose tissues from an independent set of morbidly obese female patients analyzed by quantitative real-time RT-PCR**. The real-time RT-PCR data were calculated using the relative quantification method by comparison with the expression of the housekeeping gene RPLP0 (Pfaffl, 2001). The boxes represent the medians and interquartile ranges. Whiskers represent the 10^th ^and 90^th ^percentiles. Comparisons of the groups were made with the Mann-Whitney test. Control n = 10; morbidly obese (MO) n = 31. *P < 0.05, **P < 0.01, and ***P < 0.001. Control n = 10.

To confirm the differential gene expression profiles previously observed, we analyzed gene expression by real-time RT-PCR in an independent set of morbidly obese patients. First, we chose 31 morbidly obese women, all with a BMI > 35 kg/m^2^, and 10 nonobese controls (BMI < 25 kg/m^2^). The patient characteristics are shown in Table 2. We measured the mRNA levels for CDKN1A, GADD45B, LMNA, JUN, ANGPTL4, CEBPB, CDKN2C, PCK1, SCD, and LPL in their subcutaneous abdominal adipose tissue as before (Figure 3).

**Table 2:** Clinical and biochemical characteristics of an independent set of male and female subjects

	Females (n = 41)		Males (n = 17)	
	
	Patients (n = 31)	Controls (n = 10)	Patients (n = 9)	Controls (n = 8)
Current smokers, n (%)	13 (41.9)	0 (0)*	4 (44.4)	1 (12.5)*
Age (years)	36.4 ± 10.3	49.6 ± 14.9*	37.0 ± 12.6	51.3 ± 12.4*
Body mass index (kg/m^2^)	49.5 ± 7.3	23.4 ± 3.1*	49.3 ± 7.2	23.9 ± 1.6*
Systolic blood pressure (mmHg)	128.1 ± 13.6	121.1 ± 11.7	131.3 ± 11.3	128.0 ± 8.2
Diastolic blood pressure (mmHg)	76.7 ± 9.7	66.7 ± 9.7*	83.1 ± 13.3	62.0 ± 3.8*
Mean blood pressure (mmHg)	84.7 ± 29.7	84.8 ± 9.7	88.1 ± 34.8	73.5 ± 30.1*
Serum creatinine (mg/dL)	0.79 ± 0.10†	0.76 ± 0.10†	1.15 ± 0.38	1.02 ± 0.13
Aspartate aminotransferase (U/L)	22.7 ± 9.9	17.1 ± 2.0	34.0 ± 28.0	19.2 ± 3.1
Alanine aminotransferase (U/L)	36.6 ± 32.8	14.6 ± 5.5*	61.3 ± 54.4	16.5 ± 2.8*
Fasting glucose (mg/dL)	99.5 ± 29.5	96.7 ± 8.9	120.6 ± 89.6	98.3 ± 10.7
Cholesterol (mg/dL)	190.8 ± 36.8	204.8 ± 39.9	199.6 ± 34.1	195.0 ± 19.3
Triglycerides (mg/dL)	118.1 ± 52.8	66.0 ± 20.9*	175.8 ± 99.3	104.1 ± 62.3

Data are means ± SD unless otherwise stated.

*P < 0.05 between patients and controls analyzed by Mann-Whitney U test for pair analysis after significant Kruskal Wallis test.

†*P *< 0.05 between females and males analyzed by Mann-Whitney U test for pair analysis after significant Kruskal Wallis test.

In agreement with the previous results, the expression of CDKN1A, GADD45B, JUN, ANGPTL4, and CEBPB was significantly higher and that of CDKN2C, PCK1, and LPL was significantly lower in the obese subjects than in the control women. Moreover, the LMNA and SCD mRNA levels showed similar trends to those obtained by microarray analysis, but the differences were not statistically significant (Figure 3).

PPARγ encodes two protein isoforms, PPARγ1 and PPARγ2 [22]. Both isoforms have identical sequences except for an additional 30 amino acids at the N-terminus of PPARγ2. Four major transcripts are produced from the same gene. PPARG mRNA variants 1, 3, and 4 differ only in the 5' untranslated region and encode the PPARγ1 isoform. PPARG mRNA variant 2 encodes the PPARγ2 isoform [23]. The PPARG cDNA probe used in the microarray does not distinguish the different PPARG mRNA isoforms, and the primers used to validate these results by real-time RT-PCR are located in the common transcript region of both PPARγ isoforms. Therefore, the data obtained previously (Figures 1 and 2) correspond to total PPARG expression. Because both isoforms are differentially regulated [23-25], we next used a specific set of primers for PPARγ1 and PPARγ2. As shown in Figure 3, PPARγ1 mRNA was significantly downregulated in morbidly obese women compared with the controls, whereas PPARγ2 mRNA levels did not differ between the two groups.

**Figure 4:**
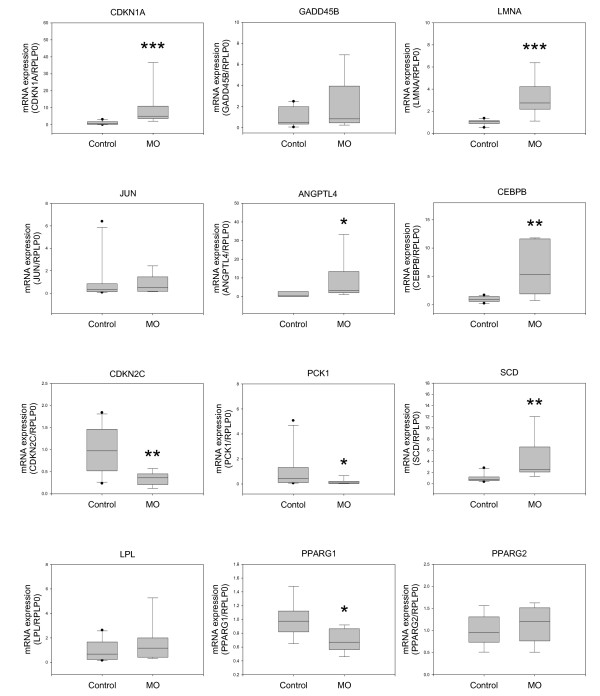
**Gene expression in the subcutaneous adipose tissues from an independent set of morbidly obese male patients analyzed by quantitative real-time RT-PCR**. The real-time RT-PCR data were calculated using the relative quantification method by comparison with the expression of the housekeeping gene RPLP0 (Pfaffl, 2001). The boxes represent the medians and interquartile ranges. Whiskers represent the 10^th ^and 90^th ^percentiles. Comparisons of the groups were made with the Mann-Whitney test. Control n = 8; morbidly obese (MO) n = 9. *P < 0.05, **P < 0.01, and ***P < 0.001.

Next, we asked whether the changes observed in the adipose tissues of obese women also occur in obese men. Therefore, we studied 9 morbidly obese men and 8 nonobese men as controls, whose characteristics are shown in Table 2. As shown in Figure 4, the expression of CDKN1A, LMNA, ANGPTL4, and CEBPB was significantly upregulated, whereas that of CDKN2C, PCK1, and PPARγ1 was significantly downregulated in the adipose tissue of male obese subjects. JUN and GADD45B mRNA levels in male patients showed similar trends to those observed in female patients, but the differences were not statistically significant. Unlike obese women, obese men had SCD mRNA expression significantly upregulated, whereas LPL expression did not differ between obese men and control men (Figure 4).

### Gene expression analysis in a mouse model of obesity

Finally, we wanted to confirm these results in a mouse model of obesity, such as the Lep^ob/ob ^mouse, first, because the sample collection procedure and the homogeneity of experimental groups can be better controlled in mice and, second, because some differences have been described in the adipocyte differentiation process between human and mouse. For this, we analyzed the subcutaneous adipose tissue from 4 female Lep^ob/ob ^mice and 4 female C57BL/6J control mice.

**Figure 5:**
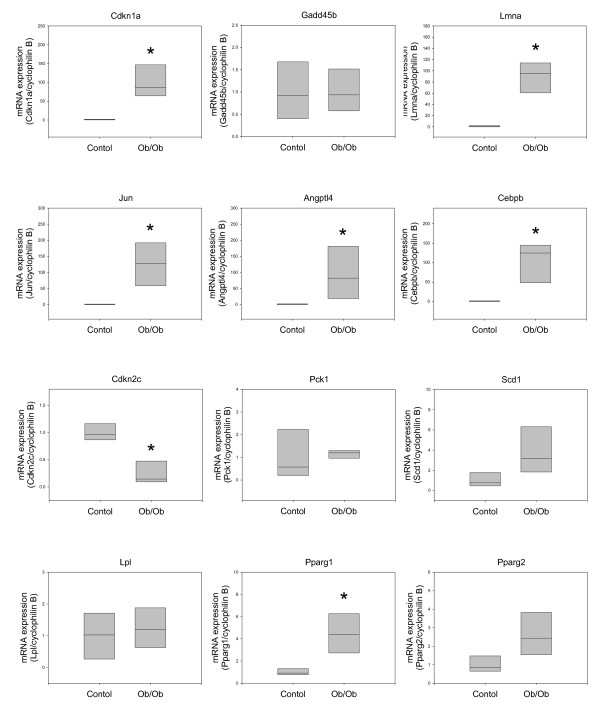
**Gene expression assessed in Lep^ob/ob ^mice by quantitative real-time RT-PCR**. The real-time RT-PCR data were calculated using the relative quantification method by comparison with the expression of the housekeeping gene CypB (Pfaffl, 2001). The boxes represent the medians and interquartile ranges. Comparisons of the groups were made with the Mann-Whitney test. Control n = 4; Lep^ob/ob ^n = 4. *P < 0.01.

As observed in the female and male obese patients, Cdkn1a, Lmna, Jun, Angptl4, and Cebpb were highly overexpressed, whereas Cdkn2c mRNA expression was highly downregulated in Lep^ob/ob ^mice as compared with control mice (Figure 5). Interestingly, and in contrast to the obese humans, the expression of Pparγ1 was significantly higher in Lep^ob/ob ^mice than in control mice, and the expression of Pparγ2 also tended to be higher in the former. Also unlike what was found in human subjects, Gadd45b, Pck1, and Lpl mRNA expression did not differ between obese and control mice. Finally, Scd1 mRNA levels were slightly higher in Lep^ob/ob ^mice than in control mice, but the difference was not statistically significant.

## Discussion

Obesity is defined as an enlargement of adipose tissue mass due to both hypertrophy and hyperplasia of adipocytes [9]. The aim of this study was to determine the gene expression profile of adipose tissue during obesity by using a focused microarray especially designed to study cell-cycle- and lipid-metabolism-related genes. We analyzed subcutaneous adipose tissue, which is the largest adipose depot in the body, accounting for approximately 80% of the total body fat. The metabolic complications associated with obesity correlate strongly with central obesity [26]. Subcutaneous abdominal fat, as a component of central adiposity, has a strong association with insulin resistance and plays an important role in the pathophysiology of obesity [6-8].

Present microarray analysis revealed that in the subcutaneous adipose tissues of morbidly obese patients the expression of genes known to be involved in adipocyte differentiation and cell-cycle control is profoundly altered. We found that the expression of C/EBPβ and JUN, which are transcription factors that regulate the first stages of adipocyte differentiation, was increased in the adipose tissue of morbidly obese patients. Conversely, the expression of PPARγ1, a transcription factor that controls the final steps of preadipocyte conversion into mature adipocytes, was reduced. C/EBPβ is not only an important regulator of adipocyte terminal differentiation since, in addition, it is a critical regulator of body weight, adiposity and tumour growth [27]. C/EBPβ deletion in Lepr^db/db ^mice reduces obesity, fatty liver, and diabetes [28]. Leptin levels are also modulated by C/EBPβ, and mice lacking C/EBPβ have severely reduced leptin levels [27]. Consistently with this, we found an increase in both C/EBPβ and leptin mRNA levels in obese patients.

In the different study subsets performed in the present work, the expression of PPARγ1 in adipose tissue was reduced in obese subjects as compared to controls, whereas no statistically significant differences were observed for PPARγ2. In Lep^ob/ob ^mice, however, both PPARγ1 and PPARγ2 were overexpressed. Although PPARγ is a well-characterized regulator of energy metabolism, the relationship between PPARγ expression and obesity is not clear. In fact, previous studies by others have produced conflicting results regarding the association between PPARγ and obesity in both humans and animals. Some studies have shown changes in PPARγ1, PPARγ2, or total PPARγ expression in subcutaneous adipose tissue [29-33], whereas others have reported no changes [30,34,35]. The different characteristics of the populations and the different methodologies used to determine PPARγ expression in these studies could explain these discrepancies. Addressing this issue, Sewter et al. also found a decrease in PPARγ1 mRNA expression in subcutaneous adipocytes from morbid obese compared with lean subjects, and a strong inverse correlation between BMI and PPARγ1 mRNA levels. In contrast, they found a significant increase in PPARγ2 mRNA expression in the morbid obese group [33]. The reduction in PPARγ1 expression in obese patients found in our study is consistent with the increase in Leptin mRNA expression also found herein, since it has been described that Leptin suppress the expression of PPARγ in adipocytes [36].

Cyclin-dependent kinase inhibitors (CDKIs) play an important role in cell cycle regulation and some of them are especially involved in adipocyte differentiation. In the present work we describe for the first time that the expression of three CDKIs is altered in human adipose tissue from morbid obese patients: CDKN1A (p21) was increased whereas CDKN2C (p18) and CDKN1B (p27) were decreased. Morrinson et al. were the first to demonstrate that these CDKIs are highly regulated in 3T3-L1 preadipocytes differentiation. Thus, p18 mRNA is expressed only during terminal differentiation, p27 mRNA is highly expressed during the whole differentiation process except in S phase, and p21 mRNA is expressed in G1 phase, decreases in S phase, and increases again at postmitotic growth arrest [14]. The importance of these CDKIs has been underscored recently in knockout mice lacking p21 and p27. Loss of one or both CDKIs, results in adipocyte hyperplasia, obesity and insulin resistance [37]. These results suggest that theses CDKIs are major regulators of adipocyte number in vivo and can have an important role in the development of adipose tissue hyperplasia during obesity. Moreover, p21 has been involved in adipocyte hypertrophy since it protected the hypertrophied adipocytes against apoptosis [38]. However, the precise contribution of these CDKIs to obesity development in humans is unclear. In this context, it is worth to note that GADD45B, a member of the growth arrest and DNA-damage-inducible gene family, also was overexpressed in the adipose tissues of morbidly obese subjects. This gene is involved in terminal myeloid differentiation and growth suppression [39], but its relationship to adipocyte differentiation has not been established. It appears, thus, that the expression of CDKIs and GADD45B, all of which regulate cell cycle and differentiation, is altered in human adipose tissue from morbid obese patients, which may reflect the relative abundance of a characteristic adipocyte subtype in fat depots from obese.

Most of our knowledge of adipogenesis is based on studies in murine-derived embryonic 3T3-L1 cells and much less in known about adipocyte differentiation in humans [40,41]. It has been established that most of the adipogenic program is similar in murine and human cells, since the expression pattern of the adipocyte differentiation-specific transcription factors C/EBPβ, C/EBPδ, PPARγ, and C/EBPα was similar in both species [15,42]. However, in contrast to murine preadipocytes, human preadipocytes do not require clonal expansion to enter the differentiation process in vitro, and they differentiate directly in response to stimulus [16]. It has been suggested that this phenomenon could reflect that adipocyte precursor cells from human adipose tissue have already undergone critical cell divisions and may be in a late stage of adipocyte differentiation [16]. In agreement with this hypothesis, it has recently been established that in humans the number of adipocytes is set during childhood and adolescence and it stays constant in adulthood [43]. However, the gene expression pattern found herein could reflect an increase in undifferentiated adipocytes as a consequence of the increased renewal rate in obese individuals. Consistently with this, Spalding et al demonstrated that obese individuals generate significantly more adipocytes per year than lean individuals [43]. Based on the relationship between adipocyte size and total body fat, they also developed a method to quantitatively estimate adipose morphology [44]. They describe that subjects can be categorized as having different degrees of either adipose hypertrophy or hyperplasia, and demonstrate that low generation rates of adipocytes are associated with adipose tissue hypertrophy whereas high generation rates are associated with adipose hyperplasia [44]. In morbid obese individuals, such as those studied herein (BMI > 35 kg/m^2^), coexist both hypertrophy and hyperplasia, however in these severe forms of obesity hyperplasia became most predominant [9,45]. It is important also to keep in mind that although the principal cellular component of the adipose tissue are the adipocytes other cellular components are also present, such as smooth muscle cells, endothelial cells, fibroblasts, and blood cells [46-48] and, thus, a contribution of these cells to this expression pattern cannot be ruled out.

Strong similarities in the expression pattern of adipogenic genes such as Jun, C/EBPβ, p21, and p18 were observed in subcutaneous adipose tissue from obese Lep^ob/ob ^mice as compared to human obese subjects. However, a major difference between both species occurs in PPARγ1 expression. Given that PPARγ1 is one of the master regulators of the adipocyte differentiation, differences in this gene may be involved in the differences observed between human and mouse adipogenesis and could be characteristic of adipocyte precursors of hypertrophic and hyperplasic adipose tissue of each species. The possibility exists, however, that the increase in PPARγ1 in Lep^ob/ob ^mice is just a consequence of the absence of leptin in this mouse model, given the role of this hormone in the regulation of PPARγ expression in adipocytes [36].

A notable finding of this work is the up regulation of ANGPTL4 expression in the adipose tissue from obese subjects, which was also found in obese mouse. This protein is a member of the angiopoietin-like family of proteins, which regulate angiogenesis [49] and may have a role in the stimulation of endothelial cell growth necessary for adipose tissue expansion [50]. ANGPTL4 is predominately expressed in adipose tissue and liver and its mRNA expression increases dramatically in the early stages of adipocyte differentiation and in the adipose tissue of diabetic (Lepr^db/db^) and obese (Lep^ob/ob^) mice [51]. More recently, ANGPTL4 has been shown to be involved in the regulation of lipid and glucose metabolism, independently of its angiogenic effects. Thus, Angptl4-deficient mice are hypolipidemic and have increased lipoprotein lipase activity [52], whereas Angptl4 adenovirus-mediated overexpression potently increases plasma triglycerides, decreases blood glucose, and improves glucose tolerance [53,54]. Hence, our results indicate that ANGPTL4 may be relevant to human obesity and, together with previous findings, point to this protein as a potential therapeutic target for obesity and obesity-related complications [54,55].

Although not consistently found in every group of obese patients of this study, the expression of LMNA is also upregulated in obesity. LMNA encodes the nuclear structural proteins lamin A and C produced by alternative splicing, which are members of the intermediate filament family of the nuclear lamina. Mutations in LMNA have been associated with a number of disorders, including familial partial lipodystrophy (OMIM 151660). Sequence variations in LMNA are also associated with greater susceptibility to the development of metabolic syndrome, dyslipidemia, insulin resistance, diabetes, and obesity [56,57]. The significance of its elevated expression in obesity remains to be established.

PCK1 gene was found to be significantly downregulated in human obese adipose tissue, whereas its expression was normal in obese mice. PCK1 encodes the cytosolic isozyme of phosphoenolpyruvate carboxykinase, which is the main enzyme controlling gluconeogenesis in the liver and kidney [58]. However, the major role of PCK1 in white adipose tissue seems to be glyceroneogenesis for reesterification of free fatty acids [59,60]. The reduction of PCK1 expression in obesity could be the consequence of the excess of triglyceride storage and the adaptation of adipocytes to get rid of some of these triglycerides throught lipolysis, perhaps mediated by glucocorticoids, which downregulate PCK1 in adipose tissue [61].

We have found sex differences in the expression of two genes involved in fatty acid utilization, such as LPL and SCD. While LPL was downregulated in female obese patients there were no differences in male obese patients. Sex differences in mRNA LPL expression and activity have been previously reported [62]. The differences between sexes were more pronounced in SCD: while SCD expression in obese female was lower than in control females, in obese males it was clearly upregulated. SCD expression is regulated by many dietary, hormonal and environmental factors that could explain the sex differences observed in our study [63]. Though, we cannot exclude the possibility that some of the sex differences were influenced by differences in the glucidic and triglyceride metabolism (Table 2).

We also found differences in other genes involved in lipid metabolism such as NPC2, ACSL1 and ACSL4, although we have not confirmed these results in an independent population of obese patients. Acyl-CoA synthetase (ACS) enzymes are essential for *de novo *lipid synthesis and fatty acid catabolism. This complex family of proteins catalyzes the activation of fatty acids necessary for their metabolism. Among these, the long-chain acyl-CoA synthetases (ACSL) activate fatty acids with chain lengths of 12 to 20 carbon atoms [64]. ACSL1 is the major form in adipocytes and it has been proposed that it mediates free fatty acid reesterification, efflux and lipid-mediated signal transduction [65]. ACSL4 is also expressed in adipocytes and it has been described that associates with lipid droplets after the lipolytic stimulation of 3T3-L1 adipocytes in vitro [66]. Interestingly, these two isoforms of ACLS presented a different pattern of expression in obese patients: ACLS1 was downregulated whereas ACLS4 was found to be overexpressed.

NPC2 is a small secreted glycoprotein that binds cholesterol and plays an important role in intracellular cholesterol trafficking [67]. The upregulation of NPC2 in obese subjects as found herein may relate with the novel role of NPC2 in adipocyte differentiation and the maintenance of the metabolic state of mature adipocytes [68]. These novel roles of NPC2 open a new perspective in the study of the adipocyte dysfunction associated with obesity that needs to be studied in more detail.

## Conclusions

Our study revealed that the subcutaneous adipose tissue of morbidly obese subjects exhibits a gene expression pattern consistent with an immature adipocyte phenotype, with augmented mRNA levels of genes involved in the early stages of adipocyte differentiation and reduced mRNA levels of genes involved in the final stages of differentiation. These findings may help to extend our understanding of the mechanisms regulating the hypertrophy and hyperplasia that occurs in obesity, and their relationship with increased adipose mass. Finally, the upregulation of ANGPTL4, found consistently in the adipose tissue of morbidly obese women and men, and of Lep^ob/ob ^mice, supports the notion that this adipokine plays a crucial role in lipid and glucose metabolism in obesity. Further investigation is required to determine the role of ANGPTL4 in obesity and obesity-related metabolic abnormalities, such as diabetes.

## Methods

### Subjects

The subjects were recruited from morbidly obese patients undergoing bariatric surgery at the Hospital Ramón y Cajal from Madrid (Spain). Extensive clinical and laboratory data were collected on each patient after his/her informed consent had been given. Patients were excluded if they presented with severe renal or hepatic failure, recent acute cardiac failure or coronary heart disease, any known malignancy, or any other condition that made them ineligible for bariatric surgery. Subcutaneous fat specimens were obtained from each subject at the time of the laparoscopic gastric bypass surgery from the peri-umbilical area. All patients were operated on by the same surgeon (R.P.) and the procedure was standardized as follows. First, the skin was cleaned and covered with special surgical drapes. After abdominal incision with cold scalpel, about 1-2 cm^3 ^(corresponding approximately to 1-2 g) of adipose tissue were removed, washed in physiologic serum, immediately snap frozen, and stored at -80°C until analysis. Control samples of subcutaneous fat were obtained from nonobese patients (BMI < 25 kg/m^2^) undergoing inguinal hernia repair surgery at the Hospital Ramón y Cajal. The study protocol was in compliance with the Helsinki Declaration and approved by the Ethics Committee for Clinical Investigation of the Hospital Ramón y Cajal (reference number: 065/03). Written informed consent was obtained.

### Mice

Eight-week-old female B6.V-Lepob mice and C57BL/6J control mice were obtained from Harlan Interfauna Ibérica (Barcelona, Spain). After two weeks of adaptation, the animals were killed, and their subcutaneous adipose tissue from the abdominal region was removed, immediately snap frozen, and stored at -80°C until the RNA was extracted. All experimental procedures were performed in accordance with the guidelines for the care and use of laboratory animals of the Animal Ethics Committee of the Hospital Ramón y Cajal.

### Microarrays

Human cDNA expression microarrays containing cDNA probes for 319 genes were produced using a SpotArray 72 spotter (PerkinElmer, Massachusetts) with TeleChem Stealth SMP3 split pins. The full-length cDNA probes were selected from the I.M.A.G.E. Consortium database and obtained from Open Biosystems. The insert cDNAs of all clones were amplified using universal primers (M13 universal forward [-21] 5'-TGTAAAACGACGGCCAGT-3' and M13/pUC reverse 5'-CAGGAAACAGCTATGACC-3') and the quality of the PCR products was routinely checked by agarose gel electrophoresis with ethidium bromide staining (E-Gel^® ^96, Invitrogen, California) and sequencing before spotting. Each PCR sample was purified with the MinElute 96 UF PCR Purification Kit (Qiagen, Valencia, California), resuspended in Pronto!™ Universal Slide Spotting Solution (Corning, NY), and spotted three times onto two different locations on Superchip I aminopropylsilane glass slides (PerkinElmer). We checked the quality of the spotting by staining one microarray with SYBR Green and one with M13-Cy5 universal primer for each batch of microarrays produced. The Lucidea Universal ScoreCard probes (Amersham-GE, Buckinghamshire, UK) were spotted three times at the beginning and end of each subarray and were used to validate the quality of the hybridizations. The microarray contained a total of 2600 spots distributed in four subarrays.

### RNA extraction

Total RNA from the subcutaneous adipose tissues was extracted using TriReagent (Sigma, St. Louis, MO, USA), according to the manufacturer's protocol. Poly(A)^+ ^RNA was purified using a GenElute mRNA Miniprep Kit (Sigma), according to the manufacturer's protocol.

### mRNA labeling and microarray analysis

The mRNA was labeled using the MICROMAX™ ASAP RNA Labeling Kit (PerkinElmer), according to the manufacturer's instructions. Typically, 500 ng of mRNA was labeled with cyanine 3 or cyanine 5 chemical-labeling reagents at 85°C for 15 min in a thermal cycler (MJ Research). Lucidea™ Universal ScoreCard™ (Amersham-GE) mRNA spike mixes were added to the labeling reactions of both the control and test samples. The combined cyanine 3 and cyanine 5 reactions were purified using CyScribe™ GFX™ purification columns (Amersham-GE) and dried in a vacuum centrifuge. The mixture was resuspended in 20 μL of hybridization buffer containing 0.4 μg/μL poly(dA) (Sigma) and 0.08 μg/μL human Cot-1 DNA^® ^(Invitrogen) and pipetted onto the microarrays. The arrays were incubated at 55°C for 16 h and then washed with ArrayIt^® ^Microarray Wash Buffers A, B, and C. The microarrays were scanned using the PerkinElmer ScanArray Express instrument with the adaptive circle method. The background-corrected data were normalized using the Gene Expression Pattern Analysis Suite v3.1 (GEPAS, http://www.gepas.org) and the global loess method [69]. Each of the six replicate spots were filtered for inconsistent replicates, merged, and log_2 _transformed with the GEPAS Preprocessing tool. The resulting data were clustered and visualized using the Mev v3.1 software. For considering a gene as differentially expressed we used a differential cut-off average log_2 _ratio value of 0.8. Pathway analysis was performed using the Ingenuity Pathway Analysis software (Ingenuity Systems, Redwood City, California, USA).

### Real-time quantitative reverse transcription (RT)-PCR

To validate microarray data, 50 ng of poly(A)^+ ^RNA was reverse transcribed using M-MLV reverse transcriptase enzyme (Promega, Wisconsin, USA) in the presence of the ribonuclease inhibitor RNAsin (2 U/μL) (Promega). Real-time PCR amplification was performed with the SYBR Premix Ex Taq kit (Takara, Kyoto, Japan) on a LightCycler 2 (Roche Applied Science, Indianapolis, USA). The initial denaturation step was at 95°C for 10 s, followed by 40 cycles of amplification at 95°C for 3 s and 60°C for 40 s. To confirm the results in an independent set of obese patients and in mice, 2 μg of total RNA was reverse transcribed as described above and real-time PCR amplification was performed on a LightCycler 480 using the SYBR Green I Master kit (Roche Applied Science). The initial denaturation step was at 95°C for 5 min, followed by 45 cycles of amplification at 95°C for 10 s, 60°C for 10 s, and 72°C for 10 s.

The melting curves were evaluated for each gene and the PCR reaction products were separated on a 2% agarose gel and stained with ethidium bromide to confirm the presence of a single product. The efficiency of the reaction was evaluated by amplifying serial dilutions of cDNA (1:10, 1:100, 1:1000, and 1:10,000). We ensured that the relationship between the threshold cycle (Ct) and the log(RNA) was linear (-3.6 < slope < 3.2). All analyses were performed in triplicate, and the relative amounts of target genes were normalized against the expression of the housekeeping gene RPLP0 (encoding ribosomal protein large P0) for human samples and against CypB (encoding cyclophilin B) for mouse samples. The primers used in the real-time PCRs are shown inAdditional file 1: Table S1.

### Statistical methods

The results are expressed as means ± SD unless otherwise stated. Clinical phenotypes were analyzed using SPSS software version 11.0 (SPSS Inc., Chicago, Illinois). The Kolmogorov-Smirnov statistical test was used for continuous variables. Logarithmic or square root transformations were applied as needed to ensure the normal distributions of the variables. The groups were compared at baseline using an unpaired *t *test or the Mann-Whitney *U *test, as appropriate for continuous variables, and the χ^2 ^test or Fisher's exact test for discontinuous variables.

For the statistical significance of the microarray gene expression t-test analysis was performed with TIGR MultiExperiment Viewer (Mev v3.1) software [70] and statistical significance was set-up at the p < 0.01 level.

Statistical analysis of real time RT-PCR gene expression was performed using Mann-Whitney test with SigmaStat software (SPSS Inc.).

## Competing interests

The authors declare that they have no competing interests.

## Authors' contributions

SRA contributed to study design, validation of gene expression and manuscript drafting. NP contributed to patient recruitment and clinical data analysis. JIB contributed to clinical data analysis and manuscript revision. NO contributed to microarray sample processing and validation of gene expression. LC contributed to sample processing and validation of gene expression. RP provided the clinical samples. DGC and MAL contributed to study design and manuscript revision. CV contributed to conception and design of the study and coordination of patient recruitment. JMB contributed to conception and design of the study, coordination, analyses of results and manuscript drafting. All authors read and approved the final manuscript.

## Pre-publication history

The pre-publication history for this paper can be accessed here:

http://www.biomedcentral.com/1755-8794/3/61/prepub

## Supplementary Material

Additional file 1 **Table S1**. Real-time PCR primers Description: Sequence of PCR primers used in quantitative real-time PCR. Click here for file
