# Supplementary material for: Gene expression profiling of subcutaneous adipose tissue in morbid obesity using a focused microarray: Distinct expression of cell-cycle- and differentiation-related genes
Source: BMC Med Genomics. 2010 Dec 23;3:61. doi: 10.1186/1755-8794-3-61 (PMC3022546; doi:10.1186/1755-8794-3-61)
Supplement: Additional file 1 — Table S1. Real-time PCR primers Description: Sequence of PCR primers used in quantitative real-time PCR. [file 1755-8794-3-61-S1.DOC]

**Table S1**. Sequence of PCR primers used in quantitative real-time PCR.
